# Serving up prevention: design and implementation of a pharmacist-led workplace diabetes prevention model for the hospitality industry

**DOI:** 10.3389/fpubh.2026.1768892

**Published:** 2026-03-26

**Authors:** Katrina P. Nguyen, Brittany A. Singleton, Carroll J. Diaz, Melissa V. Nguyen, Sharon Luc, Kambria Jeffery, Jakyria N. Booth, Airelle Harris, Brianne Herndon, Ange’l Hill, Marline Jean-Marie, Kelsie Lewis

**Affiliations:** 1 Division of Clinical and Administrative Sciences, College of Pharmacy, Xavier University of Louisiana, New Orleans, LA, United States; 2 Department of Mathematics, College of Arts and Sciences, Xavier University of Louisiana, New Orleans, LA, United States

**Keywords:** diabetes prevention, Diabetes Prevention Program (DPP), hospitality workers, pharmacist, pharmacy, pre-diabetes, pre-diabetes education, workplace intervention

## Abstract

This methods paper describes the development and implementation of a pharmacist-led, workplace-based diabetes prevention intervention tailored to hospitality workers in New Orleans. Grounded in the Social Ecological Model (SEM) and the Health Belief Model (HBM), this study integrated onsite diabetes risk screening, brief lifestyle education, provision of health-promoting tools, and facilitated referrals to affordable primary care services and CDC-recognized Diabetes Prevention Programs (DPPs). The purpose of this paper is to detail the study’s design, partnerships, logistical structure, and early process outcomes to inform others seeking to implement similar prevention strategies in underserved workforce populations.

## 1 Introduction

Diabetes remains a major public health challenge in the United States and globally. An estimated 98 million American adults have prediabetes, with approximately 80% unaware of their condition (1). Worldwide, the prevalence of type 2 diabetes has increased dramatically across all income levels over the past several decades (2). Louisiana consistently ranks among the top ten U.S. states in diabetes prevalence and lies within the “Diabetes Belt,” an area in the southeastern United States characterized by disproportionately high rates of type 2 diabetes (3, 4). Social and structural determinants including poverty, food environments, limited healthcare access, and occupational stressors, play a substantial role in shaping diabetes risk (5).

New Orleans’ economy is heavily driven by the hospitality sector, which generates approximately $10 billion annually and supports a workforce of more than 80,000 individuals (6, 7). Despite its economic importance, hospitality work is characterized by low wages, limited health benefits, irregular hours, and multiple job obligations; conditions associated with elevated cardiometabolic risk and reduced engagement in preventive healthcare (8, 9). The city’s vibrant but calorie-dense food culture further intersects with occupational and socioeconomic vulnerabilities, compounding chronic disease risk for hospitality workers (10).

Pharmacists are well-positioned to address gaps in chronic disease prevention due to their accessibility, population health expertise, and established roles in health screening, education, and referral (11–13). Pharmacist-led interventions have demonstrated success in improving diabetes-related outcomes through point-of-care testing, lifestyle counseling, and linkage to community prevention programs (14–17). However, few initiatives have extended these services into nontraditional environments such as hospitality worksites, where barriers to participation in preventive care are particularly high.

This methods paper describes the development and implementation of a pharmacist-led, workplace-based diabetes prevention intervention tailored to hospitality workers in New Orleans. Grounded in the Social Ecological Model (SEM) and the Health Belief Model (HBM), this study integrated onsite diabetes risk screening, brief lifestyle education, provision of health-promoting tools, and facilitated referrals to affordable primary care services and CDC-recognized Diabetes Prevention Programs (DPPs). The purpose of this paper is to detail the study’s design, partnerships, logistical structure, and early process outcomes to inform others seeking to implement similar prevention strategies in underserved workforce populations.

## 2 Objective

Our purpose is to present the development and implementation of a pharmacist-led, workplace-based diabetes prevention model.

## 3 Methods

### 3.1 Program design and conceptual framework

This project was guided by two complementary theoretical frameworks: the Social Ecological Model (SEM) and the Health Belief Model (HBM). The Social Ecological Model recognizes that health behaviors are shaped by multiple levels of influence, including individual factors, interpersonal relationships, workplace environments, community, and organizational structures (18). The SEM is particularly relevant for hospitality workers, whose health behaviors are impacted by irregular schedules, long hours, multiple jobs, limited access to preventive care, and workplace demands (8). By intervening directly within the worksite and building referral pathways to community resources, the program addresses barriers across several ecological levels.

The Health Belief Model was used to guide the educational and behavioral components of the intervention. HBM emphasizes individuals’ perceptions of their susceptibility to disease, the severity of potential health consequences, and the perceived benefits and barriers to taking action (19). Pharmacist-delivered education, personalized feedback from screening results, and direct referral linkages were intentionally designed to increase participants’ perceived susceptibility to prediabetes, enhance their understanding of the seriousness of progression to type 2 diabetes, and reduce practical barriers to engaging in preventive care. Providing concrete tools and guidance aimed to strengthen participants’ self-efficacy and provide a cue-to-action, which are two central constructs of HBM.

**Figure 1:**
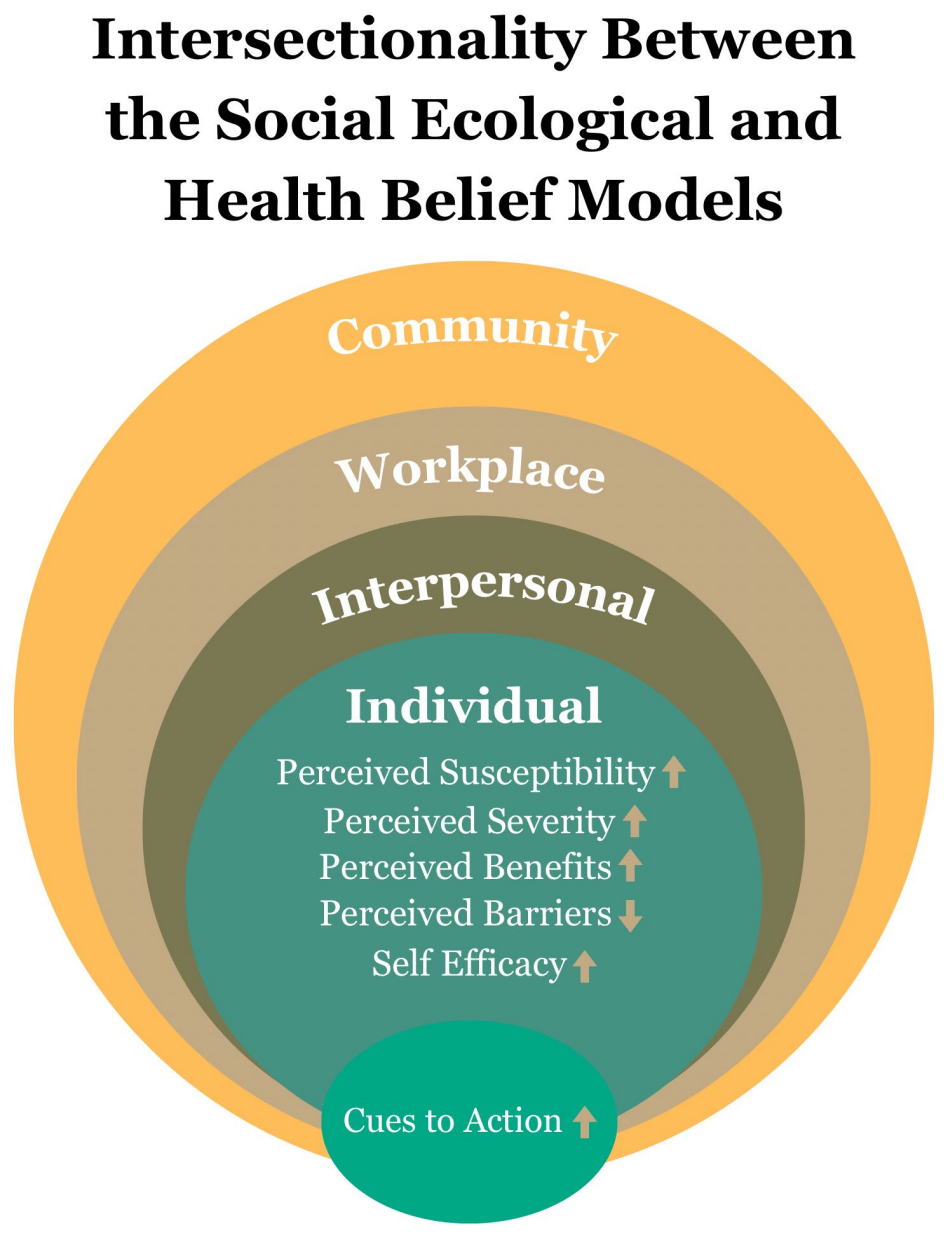
Intersection of the Social Ecological Model and Health Belief Model in diabetes prevention. Conceptual framework illustrating the intersection of the Social Ecological Model (SEM) and the Health Belief Model (HBM) in shaping preventive health behaviors among hospitality workers at risk for diabetes.

Together, the SEM and HBM provided a multilevel foundation for this intervention: SEM informed the structure and placement of the program within the hospitality workplace and surrounding community networks, while HBM informed how individual behavior change could be activated through tailored education, personalized risk communication, and facilitated access to care. The intersectionality of the SEM and HBM models is represented in Figure 1. The SEM and HBM frameworks also informed why a multi–principal investigator (multi-PI) leadership model, and diverse partnerships were essential to address the complex individual and environmental factors influencing health behaviors among hospitality workers.

### 3.2 Responsibility sharing

Given the complexity of the intervention, a multi-PI structure was essential. The project required significant coordination across multiple domains. Baseline preparation of procedures along with creation of study documents and tools precluded the study activities. Research team training required planning and leadership from the faculty PIs. Recruitment and employer engagement required establishing relationships with hospitality sites and scheduling workplace visits. Communication with external collaborators also included liaising with DPP providers and community groups. Inter-team communication was essential to carry out project activities. Screening and education logistics included organizing pharmacists, and pharmacy interns, managing on-site workflow, and ensuring protocol fidelity. Data management required overseeing survey administration, appropriate data entry, follow-up contact, data analysis and outcome tracking. Each PI contributed complementary expertise (clinical pharmacy practice, public health, community engagement, data management, and prior research experience) to ensure efficiency and high-quality execution. Frequent coordination meetings between the PIs supported decision-making.

### 3.3 Strategic partnerships

This initiative was built on a community-engaged model involving partnerships at multiple levels. Hospitality sites (restaurants, hotels, and bars) provided direct access to workers and supported on-site screening logistics. Governmental and community partners such as the city health department, non-profit healthcare advocacy groups, and a local hospitality foundation collaborated to provide access to businesses, events and connections to affordable healthcare resources. A significant partnership was forged through the American Diabetes Association (ADA) and the Diabetes Prevention Alliance (DPA). The DPA is a multi-sectoral network of partners serving Alabama, Florida, Louisiana, Mississippi, North Carolina, and Texas. These partners are committed to creating a sustainable ecosystem for the National Diabetes Prevention Program (National DPP) with a focus on providing culturally appropriate diabetes risk-reduction education access to populations at increased risk of type II diabetes (20). A formalized agreement defined this partnership, establishing DPA support for project activities, aligning the project with regional diabetes-prevention efforts and most importantly, providing connection to established DPPs who accepted at risk participants into their free prevention programs. This network-based approach allowed pharmacy professionals to serve as screeners, educators and connectors, identifying high-risk individuals, providing tailored education, and linking them to affordable, sustainable sources of preventive care.

### 3.4 Setting and participants

Investigators concentrated their efforts within the New Orleans French Quarter, a historic neighborhood and a major hub of tourism. Hospitality venues included restaurants, cafes, hotels, and bars in the area. An additional venue utilized by the investigators was a hospitality focused health fair hosted by a local marketing organization for the New Orleans tourism industry. Eligible participants included adult hospitality industry workers such as doormen, housekeeping, waiters, bartenders, chefs, etc. of restaurants, bars, and hotels. Participants were excluded if they were below the age of 18 years, reported a previous diagnosis of type I or II diabetes, were not fluent in the English language, or were pregnant if a female of childbearing age.

### 3.5 Recruitment

In preparation for participant recruitment, investigators needed to first recruit amicable hospitality venues. Investigators conducted significant outreach in the months pre-intervention to establish contact with the hospitality foundation, community organizations and individual hospitality sites. Investigators personally visited multiple hospitality businesses to discuss the project with site supervisors, general managers, or human resource representatives. Follow up emails and calls were conducted to confirm desired participation or declines. This was a continuous process until the targeted number of venues were recruited. A few locations required multiple in-person visits to confirm participation. To mitigate the impact of venue nonparticipation and broaden access to hospitality workers employed at nonparticipating sites, the research team implemented neighborhood canvassing and open community recruitment strategies. The study team conducted neighborhood canvasing by posting flyers, distributing handheld postcards, and having one-on-one conversations with hospitality workers to advertise the research events. This method increased awareness of the study among workers in the area who desired to participate in the research project, irrespective of whether their employer directly collaborated with the study team. Any interested community hospitality worker was able to access the project through participating venues. Once employer participation was confirmed, investigators began research activities at the venues. Participants were voluntarily recruited using a convenience sample method.

### 3.6 Screening

**Figure 2:**
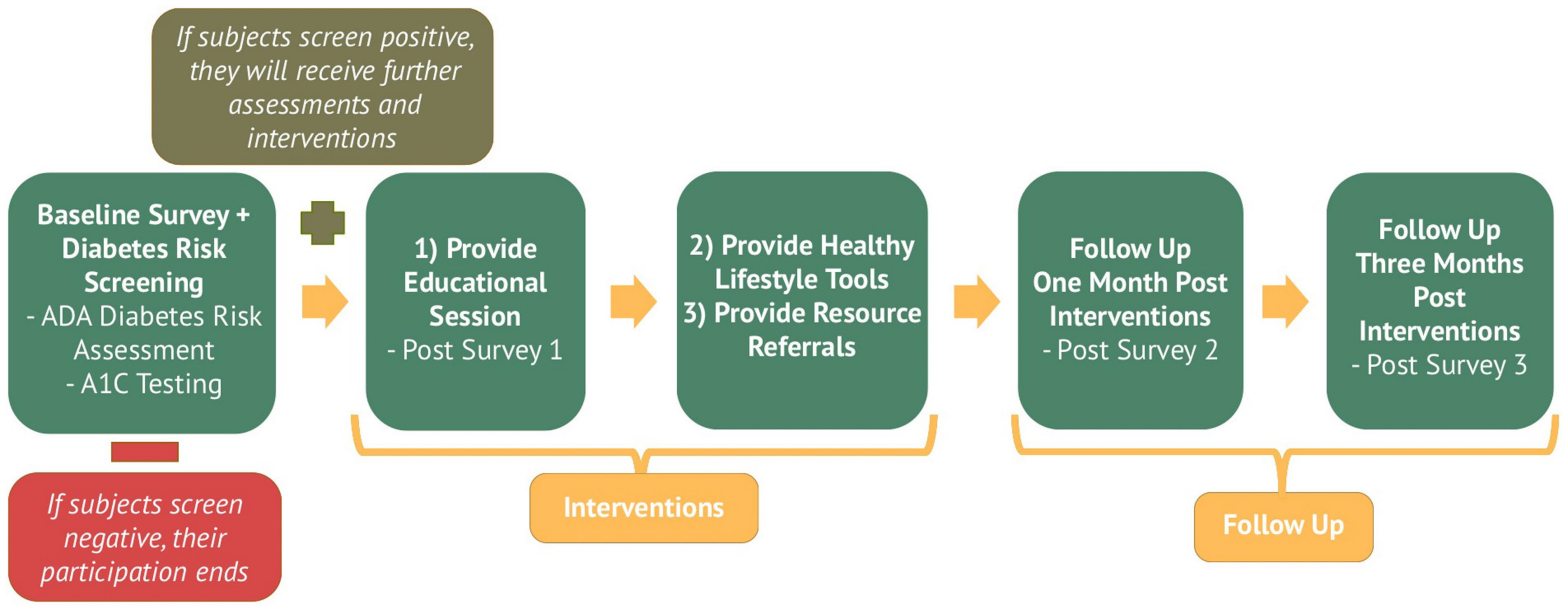
Workplace diabetes prevention interventions and study flow. Study design and intervention flow for the pharmacist-led workplace diabetes prevention program implemented among hospitality workers.

Participating venues provided space for the research activities and allowed their employees to complete the activities before, during and after their shifts. Collaborating venues permitted hospitality workers from neighboring businesses to participate in scheduled screening events in addition to their own employees. A summary of participant flow can be appreciated from the Study Design Diagram (Figure 2). At each venue, the research team established three stations to manage participant flow through the study procedures. At Station #1 participants were introduced to the study, pre-screened for eligibility, and completed necessary paperwork for registration purposes. Pre-screening consisted of verbal confirmation of age ≥18 years, employment in the hospitality industry, absence of a prior diagnosis of type I or II diabetes, English fluency sufficient to complete electronic survey materials, and confirmation of non-pregnant status when applicable. Subjects then proceeded to Station #2 which contained 3–5 screening tables set up for one-on-one screenings. At Station #2 participants were provided a tablet to electronically complete their informed consent and baseline survey. The baseline survey, which collected contact information, demographic data, employment information, health history, medication history, prediabetes knowledge and lifestyle habits, took roughly 15–20 min to complete. During the completion of the baseline survey, pharmacy student interns measured participant A1c values using the A1CNow® point of care testing system (21). Student interns also administered the 7-item ADA Risk Assessment Questionnaire to participants (22). The ADA 7-item Risk Assessment Questionnaire assigns points based on age, sex, family history, hypertension status, physical activity, and BMI, with a score ≥5 indicating elevated diabetes risk. Those who screened negative were sent to Station #3 to complete their participation. Those found to be at increased diabetes risk (via an ADA Risk Assessment score of 5 or more or an elevated A1c of 5.7 or more) continued forward to the interventional aspects of the study.

### 3.7 Intervention components

Participants found to be at risk were offered several interventions including an educational session, healthy lifestyle tools, and linkages to care. At-risk subjects were shown a brief (~5 min) education video prepared by the research team to educate them on prediabetes and diabetes prevention. The educational video, developed and scripted by the clinical pharmacist investigators, was delivered by pharmacy interns under their supervision. Specifically, the video covered prediabetes facts, healthy food choices, staying physically active, and managing stress. The video also included an introduction to the National DPP program and the benefits of joining. After participating in the education session, participants were given a 6-item posttest on the content of the session. They were also surveyed on aspects of the HBM and their intentions toward behavioral change. The 6-item post-test was developed by the research team to assess comprehension of key educational points presented in the video. Survey items assessing HBM constructs were adapted from established HBM domains. While both measures were pilot tested before use, neither were independently validated instruments. Participants were then directed to Station #3 for resources and care linkage. At Station #3, subjects received further individualized counseling on the results of their assessments from clinical pharmacists and pharmacy student interns. Subjects were then provided a diabetes prevention “toolkit” which consisted of giveaways, several educational handouts, a durable diabetes conscious portion plate, an ADA portion wheel (to reinforce knowledge of food groups and portion sizes) and a smartwatch/fitness tracking device (to track and promote physical activity). All individuals who completed the screening process through Station #3, regardless of diabetes risk status, received a $25 retail gift card as compensation for time and participation.

In addition, all at-risk participants were provided with links to further care. To ensure primary care access, each participant was given an affordable healthcare directory listing most of the Federally Qualified Health Centers (FQHCs) in the Greater New Orleans Area. The research team members then directly referred each at-risk subject to a Diabetes Prevention Program (DPP). Through membership in the ADA DPA network, the investigators collaborated with three CDC recognized DPPs. Participants were referred to a DPP according to their preference: in-person, virtual, and Spanish language speaking. For in-person class preference, participants were referred to the city department of health’s DPP. For those desiring online program delivery, a nationwide virtual program was chosen. Although English fluency was required for study participation and survey completion, a Spanish-language DPP referral option was included due to the substantial presence of Spanish-speaking hospitality workers in the Greater New Orleans region. This ensured culturally appropriate prevention access for participants who preferred program delivery in Spanish. Direct referrals were entered into an online platform by the research team members. At-risk participants’ demographic and contact information were immediately accessible by the individual DPPs. The selected DPPs later reached out to participants to enroll them into their programs according to their desire and availability.

### 3.8 Follow-up procedures

Follow-up assessed outcomes among participants identified as at-risk following the initial screening and education intervention. Follow up of at-risk participants was conducted 1-month and 3-months post interventions to assess changes in knowledge and health behaviors. Follow up data collection occurred via phone. Calls and texts were deployed as researchers made up to three contact attempts for each participant. The follow up survey specifically assessed prediabetes knowledge, lifestyle behaviors related to diet, physical activity, sleep and stress as well as perceptions and use of the healthy lifestyle tools provided and utilization of the linkages to care (seeking affordable primary care or DPP enrollment). Following successful contact and data collection, researchers re-deployed educational materials to participants via text message link. Participants were incentivized to participate in follow up by raffle entry to win one of two $50 retail gift cards provided for both the 1-month and 3-month follow up. Only at-risk participants who completed follow-up surveys were eligible for raffle entry incentives. Investigators also followed up with each DPP to confirm referrals and actual program enrollments.

### 3.9 Implementation, supplies and logistics

Implementation of the research protocol required several logistical considerations. Team composition and role delineation were important project factors. The research team was composed of two clinical pharmacist PIs, ten pharmacist interns, and a faculty statistician. The PIs were responsible for the overall design, implementation, coordination, and supervision of all aspects of the study. The PIs prepared and submitted the project for successful Institutional Review Board (IRB) approval, which required consents, collaborations, surveys, print materials, and study procedures to be prepared well in advance of the proposed study start date. They conducted oversight of survey data collection, collaborative data analysis in concert with the team statistician, and ensured study completion. All team members completed the required research training through the Collaborative Institutional Training Initiative (CITI) training platform. Due to the nature of the study procedures, this human subject research was classified as a clinical trial, and a clinicaltrials.gov entry was created by the investigators. Student pharmacists were integral members of the team and were involved in several aspects of the research activities including recruitment material development, participant registration, eligibility screening, survey facilitation, A1c testing under supervision, ADA Risk Assessment administration, delivery of educational materials, data entry, and referral coordination. Interns also, organized supplies, trained and rotated positions between Stations #1–3 to become proficient in all three station duties. Interns also conducted follow up surveys via phone. The clinical pharmacist PIs provided guidance to student interns throughout the study period, serving as research preceptors and mentors. Community partners at the hospitality workplaces were critical project collaborators. Management at each workplace assisted in ensuring that space was allocated for the research activities and allowed employees to participate in the study, although participation remained completely voluntary.

In this study design, several arrangements were made regarding required supplies including supply design, procurement, organization, transportation, and storage. Supplies specifically tailored to the intervention, such as the durable portion plate, were pre-designed in collaboration with the manufacturer. Flyers and postcards were also developed prior to recruitment activities and required printing services. All items needed for the screenings, surveys, and interventions were obtained beforehand. Multiple electronic tablets were purchased for participants to complete consents and the web-based surveys. A cellular phone was also purchased to create a central mobile number for parties to communicate with the research team. Basic supplies such as tablecloths, clipboards, table markers, tape, pens, handouts, and accordion file folders were purchased. Hemoglobin A1c screening required the purchase of medical supplies such as the pts. Diagnostics A1CNow® point of care testing kits. These point of care testing kits require room temperature storage (64 °F to 82 °F or 18 °C to 28 °C), which necessitated temperature-controlled use, transport and storage. Point of care testing also required a Clinical Laboratory Improvement Amendments (CLIA) Waiver Certificate, which was obtained through the college’s Wellness Center. Additional required medical supplies included alcohol swabs, gauze pads, band aid, sharps containers, hand sanitizer, paper towels, surface sanitizing wipes, supply bins, and trash bags. Participant giveaways and healthy lifestyle tools such as durable portion plates, fitness trackers/smartwatches, portion wheels tote bags, water bottles, lip balms and handouts were provided and ordered ahead of the intervention. Large foldable rolling wagons were purchased to transport these supplies to and from sites or vehicles.

### 3.10 Data management and analysis

To ensure confidentiality, multiple safeguards were implemented. All study data were managed using a combination of secure storage forms and Health Insurance Portability and Accountability Act (HIPAA) compliant digital systems. Qualtrics served as the primary platform for data collection. Documentation for all survey instruments, and participant responses were stored in its encrypted environment. Investigators monitored survey responses to ensure data quality and rectify any discrepancies due to participant or surveyor error. Upon completion of data collection, Qualtrics data were exported into a password-protected Excel file accessible only to the study PIs. All electronic files were stored on password-protected institutional computers, and all analytic datasets were fully de-identified prior to analysis.

Data access was restricted to trained members of the research team and the three DPP partner organizations accepting participant referrals. DPP referrals were initiated using the ADA’s online referral platform, allowing connection to the participant’s preferred DPP delivery format (in-person or virtual) while protecting personal health information. Follow up communication with DPPs confirming participant enrollment utilized password protected excel files via organizational email. The study PIs maintained oversight of all data management procedures to ensure continued compliance with HIPAA and institutional security standards.

International Business Machines (IBM) Statistical Package for the Social Sciences (SPSS) Statistics version #29 was used for statistical analysis. Data were imported into SPSS after the removal of all identifiers. Descriptive percentages were generated for demographic and other pertinent variables. Pre-test and post-test data derived from baseline and follow up survey responses were analyzed using paired t-tests. Repeated measures comparisons were conducted to compare baseline health behaviors to follow up responses. Additional comparisons of variables between groups were completed using chi-square analysis.

## 4 Results

Participant recruitment, screening, and interventions were conducted from June to July of 2025. A total of 17 hospitality worksites were directly approached by the investigators for collaboration. Seven hospitality venues agreed to participate in the research study and 10 venues declined participation or were nonresponsive. Participating venues included one hotel, five restaurants/bars, and one hospitality event venue. Worksite partnership attempts were bipolar in nature. Collaborating employer partners were very accommodating to project needs, often providing space, setup assistance, and refreshments. Those worksites not interested were generally not responsive to communications. However, feedback from responsive decliners cited barriers including concerns about workflow disruption, limited available space, liability concerns, and competing operational priorities. Across the seven participating sites, the pharmacy team conducted 10 screening events over the study period. Events ranged in time from 3 to 10 h long with an average timeframe of roughly 6.5 h duration. A total of 303 eligible individuals completed Station #2 screening procedures and were included as study participants, with 63% of participants being employees of participating venues, and the remainder being employed at neighboring hospitality establishments. Of these screened participants, 114 (38%) met study criteria for increased diabetes risk (elevated A1c or ADA Risk Assessment score). All 114 (100%) at-risk participants were referred to a partner DPP using the ADA’s online referral system.

The project established and maintained active communication with 3 DPP providers, facilitating referral coordination. Of the submitted referrals, 111 (97%) were confirmed as successfully received by partner programs. Three referrals were not initially received due to a data transfer error within the receiving DPP’s workflow, which resulted in loss of participant contact information. The issue was subsequently identified and corrected to facilitate contact with those three participants. Based on DPP delivery partner feedback, 76 (68%) of the received referrals declined enrollment or were unreachable, 25 (23%) expressed interest in the programs, but were waitlisted due to availability or preference, and 10 (9%) enrolled into a DPP program. Follow up data collection occurred via phone survey from July to October of 2025. Of the 114 at-risk participants, 40 (35%) and 20 (18%) completed the 1-month and 3-month follow-up surveys, respectively. Of 114 at-risk participants, 12 (11%) completed both follow-up surveys. A total of 10 student pharmacists were trained and completed screening, education, referral and follow up procedures. Qualtrics survey completion rates were high, with 100% of surveys fully documented in the system.

The intervention demonstrated moderate operational feasibility in real-world hospitality settings as evidenced by successful employer partnerships, consistent protocol delivery across 10 screening events, and 100% referral initiation among at-risk participants. The average time required to complete a full initial screening encounter (baseline consent and survey, risk assessment, point-of-care A1c, education, and referral navigation) was approximately 20 min, allowing research staff to operate efficiently within busy and variable workplace environments. Participant feedback indicated high acceptability of pharmacist-led screenings and expressed gratitude for the interventions delivered at their workplace. Several hospitality venues requested additional or annual visits, emphasizing the perceived value of onsite prevention services for their employees. DPP partners reported difficulty contacting several referred participants. However, considering those they were able to contact, partners reported that pharmacy team-generated referrals were well educated on the importance of diabetes prevention, and were interested in joining the DPP, highlighting the successful counseling performed by the research team and supporting the success of this cross-sector collaboration.

Participants were 41 years of age on average, with 50% identifying as women and 74% identifying as racial or ethnic minorities. Most (60%) participants worked in restaurants and bars. Baseline health indicators reflected elevated health risk with 28% of participants reporting a family history of diabetes and 23% reporting a diagnosis of comorbid high blood pressure at the time of screening. Participants had an average BMI of 27.5 ± 5.7 kg/m^2^ and 65% had BMI ≥ 25 kg/m^2^. Participants had an average A1c of 5.5% (SD = 0.64). Fifty percent of participants had publicly funded health insurance or were uninsured. These descriptive findings illustrate the moderately high-risk profile of hospitality workers engaged through the intervention.

## 5 Discussion

This methods paper describes the development and early implementation of a pharmacist-led, workplace-based diabetes prevention intervention tailored to hospitality workers in New Orleans. This population faces pronounced socioeconomic, occupational, and structural barriers to chronic disease prevention. The intervention successfully integrated multilevel strategies informed by the Social Ecological Model (SEM) and Health Belief Model (HBM) by situating screening and prevention activities directly in hospitality worksites, providing personalized risk counseling, and offering linkage to sustainable diabetes prevention resources. Cross-sector partnerships among academic investigators, employer partners, governmental agencies, non-profit organizations, and ADA-affiliated DPP providers enabled coordination from screening through referral. Key findings show that (1) employer partnerships were feasible in a subset of hospitality venues, (2) pharmacists and student pharmacists efficiently administered screenings, education, and referrals in the workplace, and (3) more than one-third of screened participants met criteria for increased diabetes risk. These results emphasize both the need for and practicality of delivering prevention services in this occupational setting, where traditional access to care is limited.

The process proved adaptable to diverse hospitality venues including restaurants, bars, hotels, and event spaces. High acceptability among participants and return requests from employer partners suggest strong receptivity to pharmacist-led services in the hospitality environment. Despite challenges with follow-up response and DPP enrollment, the intervention successfully delivered immediate counseling, health-promoting tools, and referral pathways to CDC-recognized Diabetes Prevention Programs (DPPs).

### 5.1 Relation to similar studies and importance of findings

The findings of this study align with a growing body of literature demonstrating the essential role of pharmacists in chronic disease prevention and public health. National organizations, including the American Society of Health-System Pharmacists and the CDC, have long recognized pharmacists as accessible, trusted providers capable of delivering screenings, counseling, and prevention services across diverse community settings (12, 23). Multiple studies have shown that pharmacist-led interventions improve glycemic outcomes, blood pressure control, and preventive health behaviors, particularly among underserved or minority populations facing significant structural barriers to care (14, 24–26). The present findings reinforce these trends by demonstrating that pharmacists can effectively extend prevention services into nontraditional environments such as hospitality worksites, which are settings characterized by irregular schedules, high job demands, and limited healthcare access. While prior studies have documented the effectiveness of community pharmacy-based diabetes prevention efforts, few have focused specifically on occupational groups such as hospitality workers, who experience distinct socioeconomic and environmental risks (14–17, 27, 28). By embedding screening and education directly within familiar workplace settings, this model addresses logistical constraints identified in workforce health literature and aligns with evidence supporting place-based approaches to preventive care (29, 30). Moreover, the integration of direct referrals to CDC-recognized Diabetes Prevention Programs represents an innovative extension of pharmacist-led services, creating a crucial pathway from risk identification to evidence-based intervention. The high prevalence of diabetes risk observed among participants highlights the urgency of such targeted prevention strategies and underscores the importance of adapting public health interventions to populations that have been historically underrepresented in prevention research.

The clinical relevance of this study lies in its demonstration that pharmacist-led initiatives can identify high-risk individuals who might otherwise remain undiagnosed or disengaged from preventive services. Workplace-based diabetes risk screening eliminated major barriers such as transportation, cost, and scheduling, issues well documented in workforce health literature (30, 31). Tailored counseling grounded in HBM constructs, combined with distribution of concrete health-promoting tools (e.g., fitness trackers, portion plates), provided immediate cues to action and practical strategies for behavior change.

By directly referring at-risk individuals to DPPs, a rigorously validated program known to reduce diabetes incidence by up to 58%, this model bridges the often-missing link between screening and long-term prevention (32). When implemented at scale, similar models may improve early detection of prediabetes, increase DPP participation, and ultimately reduce diabetes incidence in high-risk occupational groups.

This study had several notable strengths. It leveraged two complementary theoretical models (SEM and HBM) to address both individual and structural determinants of diabetes risk, resulting in a multilayered intervention delivered in the workplace, an underutilized setting for chronic disease prevention. Pharmacists played a central role as frontline public health educators in this intervention, efficiently conducting all components of the intervention from screening to referral and follow up. Beyond conducting screenings, clinical pharmacists and interns interpreted A1c values within ADA-defined thresholds and were responsible for the critical components of educational content development, risk communication, and individualized counseling. Pharmacists receive formal training in patient communication, motivational interviewing, chronic disease prevention, and medication management. As trusted and accessible healthcare professionals, their role uniquely positions them to educate diverse community members and guide patients’ informed health decisions. The clinical pharmacy research team effectively translated risk information into understandable and culturally appropriate language, delivering structured counseling tailored to each participants’ results. In this workplace setting, pharmacists bridged risk identification with actionable prevention strategies. Additionally, strong partnerships with hospitality worksites, local organizations and the ADA Diabetes Prevention Alliance created a coordinated pathway from risk identification to prevention program enrollment. The distribution of health tools further reinforced behavioral messaging and provided practical support for immediate lifestyle changes.

However, limitations should be acknowledged. Employer engagement was variable as less than half of contacted venues agreed to participate, limiting generalizability and reach. The intervention was labor-intensive and required substantial personnel for onsite setup, multi-component screenings, and follow-up communication. Follow-up survey response rates were lower than desired but were near to existing response rate estimates (33). Actual DPP enrollment lagged despite high referral rates, suggesting persistent barriers such as competing job demands and limited time. To reduce the number of calls to participants, eliminate delay and streamline the enrollment process, on-site DPP enrollment rather than referral may be a helpful strategy to employ in future investigations. The use of an OTC A1c device introduced potential measurement variability and required strict temperature controls. Additionally, workspace layouts at some venues may have limited participant privacy.

Future research should evaluate strategies to expand scalability and improve long-term engagement with DPPs. A mobile unit equipped with clinical supplies and staffed by healthcare professionals may enhance portability of screenings and allow prevention services to reach populations with limited access to traditional healthcare settings. Broader implementation across the Greater New Orleans region through partnerships with institutional wellness centers, community pharmacies, and employer wellness programs may increase reach and sustainability. Replication of this model within other Diabetes Prevention Alliance partner states may further evaluate reproducibility across diverse workforce and community contexts.

Longitudinal studies are needed to examine outcomes such as behavioral change, A1c trends, DPP enrollment and retention, and diabetes incidence. Comparative effectiveness studies could assess pharmacist-led interventions delivered in workplaces versus community pharmacies or mobile sites. Additional research should explore mechanisms to improve follow-up engagement, including text-based reminders, employer-supported time allowances, or integrated digital health tools.

## 6 Conclusion

A pharmacist-led, workplace-based diabetes prevention model is both feasible and impactful for reaching at-risk hospitality workers; an underserved population facing substantial barriers to preventive care. Integrating behavioral theory, strategic partnerships, onsite screenings, tailored counseling, practical lifestyle change tools and direct DPP referrals provides a replicable framework for expanding chronic disease prevention in nontraditional settings. Ongoing refinement and broader implementation have the potential to advance public health outcomes in communities with elevated diabetes risk.

## Data Availability

The datasets presented in this article are not readily available due to participant confidentiality. Requests to access the datasets should be directed to Brittany Singleton, PharmD, bsingle2@xula.edu.
